# Study protocol: a pragmatic trial reviewing the effectiveness of the TransitionMate mobile application in supporting self-management and transition to adult healthcare services for young people with chronic illnesses

**DOI:** 10.1186/s12913-022-08536-8

**Published:** 2022-11-29

**Authors:** Jeffrey Yeung, Yisselle I. Virella Pérez, Shehani C. Samarasinghe, Vhari Forsyth, Vathsala Agarwalla, Katharine Steinbeck

**Affiliations:** 1 The Clinical School at The Children’s Hospital Westmead, Specialty of Child and Adolescent Health, The Faculty of Medicine and Health, The University of Sydney, Sydney, Australia; 2 The Academic Department of Adolescent Medicine, The Children’s Hospital Westmead, Sydney, Australia

**Keywords:** Adolescent, Chronic illness, Transition, Self-management, Mobile application, Pragmatic trial

## Abstract

**Background:**

Transition from paediatric to adult heath care services is a challenging time for many adolescents with chronic illnesses and may include deterioration in illness control as a consequence of inadequate self-management skills, poor understanding of their chronic illness and failure to engage with adult services. Successful transfer of health care requires the development of self-management skills and increased autonomy. Mobile technology has been proposed as a modality to assist this process. Evidence is limited and generally restricted to illness specific applications. The TransitionMate app (TMApp) is a generic (non-illness specific) mobile application designed to support young people with chronic illness in their transition from paediatric to adult health care services. The overall aim of the study is to assess the effectiveness of TMApp in improving engagement and retention of adolescents with chronic illness within adult healthcare services, as well as preventing the deterioration in illness control and unplanned hospitalisations.

**Methods:**

The TransitionMate trial is a dual centre, pragmatic, single arm, mixed methods cohort study conducted within two university teaching tertiary paediatric hospitals in Australia. Data collection points are planned at 0, 6, 12 and 18 months. Outcome indicators include: usage of TransitionMate, engagement with adult services, quantitative markers of illness control, and unplanned hospital admissions. Data are collected through telephone interviews with the participants, their primary healthcare providers, electronic medical records and de-identified mobile application analytics. The development of the application involved co-design with recently transitioned young people with a number of chronic illnesses as well as online user experience in younger adolescents.

**Discussion:**

The TransitionMate study is the first identified trial of a generic mobile application designed to support adolescents with chronic illnesses during the transition process. Results are expected to provide novel insights into the value of technological tools in the transition space, especially their effectiveness in improving both the transition process and clinical outcomes of adolescents with chronic illnesses. Furthermore, the approach of a pragmatic study design may help identify research methods better designed to overcome inherent challenges in research involving adolescents, transition of care and use of mobile application technology.

**Trial registration:**

Registered retrospectively as of 30/1/2020 with Australian New Zealand Clinical Trials Registry: ACTRN12620000074998.

**Supplementary Information:**

The online version contains supplementary material available at 10.1186/s12913-022-08536-8.

## Value of a pragmatic trial in studies involving

- Transition of care in chronic illness
- Adolescents
- Effectiveness of a mobile application designed to change behaviour

## Introduction

### Background and rationale

Chronic illnesses are long term conditions that if left untreated or incorrectly managed, may cause numerous complications that seriously affect overall long term health and wellbeing. To maintain optimal physical health, a young person with a chronic illness needs to perform repetitive and specific illness management tasks and often maintain a lifestyle with regimentation and restricted conditions. For young people with chronic childhood illnesses, expectations around self-management often change dramatically as they transition from paediatric to adult care [1, 2].

Transition refers to the planned movement of adolescents or young adults with chronic illness from paediatric centred care to adult health care systems [3]. This process requires the young person to develop increased autonomy and independence from parents/guardians. Young people also have to adjust to care from new clinicians in adult healthcare services, where the management approach is more individual with expectations of full autonomy, and less family-oriented [4, 5]. Additionally, during this transitional phase, the treatment itself may change or require modification, representing an additional challenge. During this unique life stage of emerging adulthood, young people also need to incorporate their illness management processes into other major transitions such as leaving school or home, entering employment or tertiary studies and entering new relationships [6–8]. It is during this time that there is often deterioration in illness control and even unplanned hospitalisations as a result of inadequate self-management and failure to respond to physical alerts of illness deterioration [9–11].

Another important factor to consider in chronic illness self-management of young people is the influence of psychological well-being, with Adam and colleagues in the United States in 2019 reporting the adjusted risk of incidental mental health conditions being 51% greater in youth with chronic physical conditions when compared with those without [12–15]. Therefore, with the complexity of the transition process and its role in shaping the future health and lives of these young people, it is imperative that the health care system continues to find new ways to motivate and support young people to improve their chronic illness self-management. The purpose of this study is to investigate how new digital technology can be used to support environments where young people are focused on attaining autonomy in many aspects of their lives.

Mobile technology is widely used by adolescents and young adults, creating a unique opportunity to explore innovative ways of communicating and supporting young people with their chronic illness self-management during the transition to adult health care systems [3, 12, 16]. To date, there is limited evidence regarding use of mobile phone applications aimed at supporting self-management in adolescents living with chronic illnesses, with the available ones generally being disease specific applications.

Majeed-Ariss and colleagues in 2015 conducted a systematic review to examine the literature (between 2003 to 2014) regarding the effectiveness of mobile apps designed to support adolescents in the management of their physical chronic conditions [17]. A key conclusion from the study was the significant lack of evidence-based apps in this field, highlighting the need for further investigations and research. A meta-analysis drawing on data published between 2006 and 2016 examined the use of mobile health interventions in improving health outcomes in young people [18]. The study showed that digital technology can be effective in eliciting meaningful improvements in paediatric health behaviour and associated health outcomes. However, the average age of participants was 11.4 years, and many of the papers focused on interventions targeting care givers, or on immunisation adherence, thereby limiting conclusions around adolescents’ self-management with chronic illnesses.

A recent systematic review conducted by Virella Pérez and colleagues examined the literature published from 2013 to 2018 on the utility and effectiveness of mobile and/or web-based health applications that support self-management and transition in young people with chronic physical illnesses and identified that apps were invariably illness specific [19]. The lack of literature and clinical data remains a key theme and the review was unable to provide evidence for effectiveness of this approach. However, the review did provide insights into future study design with reference to the development, evaluation and effectiveness of applications tailored for adolescents with chronic illnesses, including the involvement of adolescents in such designs [19, 20]

Traditional models of healthcare, based on face-to-face meetings and information exchanged directly between the patient and the healthcare professional, are no longer the only communication avenue and approach. The ubiquitous nature of mobile devices offers an opportunity to explore new means of interacting with young people over the management of their chronic condition in the period between when they leave paediatric health care and finally engage with adult healthcare. For example, mobile phones and applications can be used for data management, and a reminder system for self-management tasks and appointments. Applications could also serve as a reporting tool for information such as a patient’s emotional well-being over time, as well as real time data on events, problems, thoughts or feelings almost anywhere, anytime. Much of this information can be remotely extracted as the digital footprint [21, 22]. This digital footprint, when applied appropriately, may be a valuable tool in understanding and improving the healthcare experience of patients, including during the transition process.

Despite the potential benefits of mobile applications, there is a significant void in generic evidence based mobile tools to support adolescents with chronic illnesses in transitioning from paediatric to adult healthcare. Furthermore, despite the intuitive assumption that young people would readily use these media in their healthcare management, there is a lack of rigorous evaluation and empiric research data to provide evidence-based support for this assumption. The conclusions from literature reviews, along with the growing need for innovative and effective tools to support the transition process for young people, have led our investigators to create a purpose-built mobile application called TransitionMate. This mobile application was designed to be a generic tool to support young people with a chronic illness during their transition from the paediatric to adult healthcare system. The application aimed to provide some support at a time when young people may be resisting assistance from family as a mark of independence, and particularly between the last paediatric and first adult health are visits.

The lengthy development of, and technical challenges of the TMApp, together with analytics, will be submitted as a separate manuscript. The application we describe in this manuscript is the final product for clinical trialing.

Our study aims to evaluate the effectiveness of a mobile health intervention (TMApp) in supporting self-management and transition to adult healthcare services for young people with chronic illnesses.

### Objectives

#### Primary objective

- To examine the effectiveness of the TMApp on rates of engagement with adult services in young people with chronic illness (such as type 1 diabetes mellitus, inflammatory bowel disease, cystic fibrosis, mixed connective tissue disorders) transitioning from paediatric to adult care.

#### Secondary objectives

- To examine the rates of retention with adult services at 12 and 18 months in young people with a chronic illness who have successfully transitioned (i.e. successfully engaged) to adult services.
- To understand how disease control changes during the first 18 months post transition
- To understand if the use of the TMApp can assist with preventing deterioration of chronic illness control in young people in the 12 months post transition.

### Trial design

The trial is designed to be a two centre, pragmatic, single arm, mixed methods cohort study. As this is a single arm study, there will be no randomisation or allocation process on recruitment. Results will be compared with existing historical data from our previous research as well as others.

Source of historical data from the following source:

1. Perry L, Steinbeck KS, Dunbabin JS, Lowe JM. Lost in transition? Access to and uptake of adult health services and outcomes for young people with type 1 diabetes in regional New South Wales. Med J Aust 2010 Oct;193(8):444–9.
  Audit of 239 young adults aged 18–28 years with type 1 diabetes accessing five adult diabetes services before 30 June 2008 in three geographical regions of New South Wales [23]
2. Garvey KC, Markowitz JT, Laffe LMB. Transition to Adult Care for Youth with Type 1 Diabetes. Curr Diab Rep 2012 Oct;12(5):533–541 [24].
  Data were collected between August 2004 and October 2005 through face-to-face interviews with over 200 clinicians in 68 clinical services in tertiary paediatric hospitals in New South Wales, providing information on approximately 4200 patients

### Explanation for the choice of comparators

Based on the number of patients which fits the eligibility criteria within the hospital network each year, and the anticipated recruitment based on previous adolescent studies of 1:4 approached actually consenting, it was unlikely that adequate power would be achieved if we were to recruit for a comparator group within the resourced time frame available. The necessary updates and debugging in mobile technology and their cost, coupled with the uncertainty of success based on previous literature guided the choice of a single arm study. It was important that we had more knowledge about the TMApp’s utility and durability of use before progressing to a comparator study.

## Methods: participants, interventions and outcomes

### Study setting

Participants will be recruited from the paediatric outpatient clinics of the two tertiary paediatric hospitals within the Sydney Children’s Hospital Network - Sydney Children’s Hospital at Randwick and The Children’s Hospital at Westmead - which are both located in Sydney, Australia.

Suitable patients aged 16 years and over with a chronic illness requiring transition to adult services and who are in their final year of paediatric care/attending their penultimate paediatric appointment will be identified by the treating team or clinic staff and will be approached for recruitment in the study by a member of the research team. Recruitment in the penultimate appointment is to reduce the risk of any paediatric intervention confounding the effect of the mobile application.

### Eligibility criteria

#### Inclusion criteria

- Current patients of the Sydney Children’s Hospital Network who have a chronic physical illness (defined as a long term physical health condition that is likely to have consequences for the overall wellbeing of a patient if inappropriately managed or left untreated).
- 16 years of age and over
- Transitioning to adult healthcare services within the next 12 months
- Consent to use the TransitionMate app and follow-up for 18 months

#### Exclusion criteria

- Intellectual disability affecting use of mobile phone application
- Primary diagnosis of mental health disorder
- No access to personal smartphone

### Who will take informed consent?

Patients from subspecialty outpatient clinics at Sydney Children’s Hospital or The Children’s Hospital at Westmead who are considered appropriate by their clinical team will be approached and consented in person by a research investigator. Adolescents and their parent/guardians will be provided with an information sheet about the study. If the young person is 18 years of age they will be able to sign the consent form independently. If they are aged between 16 to 18 years of age, our institutional Human Ethics Research Committee (HREC) requires a parent/guardian signature for consent. Written consent (Additional file 1-Patient informed consent form) will be obtained from all participants.

### Interventions

#### Intervention description

##### Definitions

- Application – TransitionMate mobile application
- Engagement – At least one attendance to the adult service in the preceding 6 months at the 6 months follow-up.
- Retention – At least one further attendance to the adult service in the preceding 6 months at 12 and 18 months follow-up.

##### Application development

The TMApp was initially designed through a collaboration between The School of Electrical and Information Engineering, The University of Sydney, Australia, The Academic Department of Adolescent Medicine at The Children’s Hospital at Westmead, Sydney, Australia and the Agency for Clinical Innovation, one of the pillars of New South Wales (NSW) Health, NSW, Australia. The app was designed to support self-management and transition from paediatric to adult care in young people with a chronic illness. It allows young people to develop self-management skills by: setting reminders for treatments required, medications or appointments. The six key features are: To do list; Contacts; Measures; Images; Medications; and Moods which are described below. These functions increases accessibility to important healthcare information for the young people as long as they have their mobile phones with them. Furthermore, they are able to email relevant information to care providers as required.

The TMApp requires the young person to input their own health related data, and we anticipate the initial set up of the app and input of health related data to take no more than 45 minutes. All health related data are stored locally on the patient’s device and do not get uploaded onto a server. Nor is it communicated with health care providers. The research team will be able to obtain a ‘digital’ footprint of all the participants’ interactions with the app. This will be de-identified group data which allow the research team to understand how frequently the app is used, which functions are most used, and how much time is spent on the app and each function. However, there is no ‘tracking’ of individual patient information in the sense that none of the information the young person has input into the app is relayed back to the app developer/health professionals or anybody else including the developers who provide the data. Patients are free to share their information with relevant health professionals if they so choose.

##### Intervention: downloading and use of the TransitionMate mobile phone application to assist transition of adolescent from paediatric to adult clinic services

The TransitionMate mobile phone application will be downloaded onto the participant’s mobile phone once the study has been explained and consent has been obtained. Download is performed by the participant under the guidance of the researcher. The download will be available from the Apple Store (ios) or Google Play Store (android). Use of the application would involve the participant independently opening the mobile application and using the features within. These features are:

- *To do list*
- *Measures of illness control*
- *Contact list*
- *Medication Lists*
- *Images* (Take and save photos)
- *Mood tracker using words or emojis*

The number of times the intervention (TMApp) and the features are used is determined by each participant. TMApp usage will be reviewed at the 6, 12 and 18 months by follow up phone calls (Additional files 4, 5, 6). These self-report data can be broadly compared to the application data analytics, which are cohort only data. Up to 5 attempts will be made to contact each participant via a telephone call at each of the 6, 12 and 18 months’ time points. A SMS message will be sent if the call is not picked up with contact details provided for a return call when it is convenient for the participant. Participants will no longer be contacted should they express at any time that they would not like any further communication with regards to the study.

### Other data collection

The following data will be collected at baseline: gender, date of birth, ethnicity, diagnosis, young person’s email and mobile phone number, and one parent’s contact number and email (Additional file 8). We will record the standard transition care which the young person has received. Established transition services in our patient population includes ACI Transition Care Service, Trapeze Transition Service [25, 26] and joint paediatric and adult clinics. The NSW Health Agency for Clinical Innovation (ACI) Transition Care Service is a state-wide care coordination service responsible for supporting young people (aged between 14 and 25 years) with chronic illness/disability as they move from children’s health services to adult health services. Trapeze is part of the Transition Care Network and provides a supported transition into adult care for patients aged 14–25 within the Sydney Children Hospital Network. We will also obtain the details of the adult service that the treating paediatric team has referred the young person to. Included in the consent is collection of EMR data on measures of illness control and frequency of visits to the paediatric service in the previous 12 months.

Table 1 shows a sample list of conditions and relevant illness markers compiled in consultation with on the Clinical Services which have agreed to take part in the study. If other conditions not mentioned here are included, the research team will determine the preferred control marker in consultation with the respective clinical team.

**Table 1:** Sample list of illness markers for chronic illnesses

Chronic illness	Illness marker
Acquired brain injury	Functional Independence Measure (FIM), the Glasgow Outcome Scale (GOS), and the Disability Rating Scale (DRS)
Autoimmune thyroid disease	Free T4; Free T3; TSH
Congenital Adrenal Hyperplasia	17 OHP; Plasma Renin Ratio; Testosterone, Androstenedione, Dehydroepiandrosterone sulfate (DHEAS)
Cystic fibrosis	FEV_1_; Weight
Duchenne muscular dystrophy	Creatinine kinase; Lung function test; GMFCS
Epilepsy	Number and frequency of seizures
Inflammatory bowel disease - Crohn’s, Ulcerative colitis	Weight; CRP; Faecal Calprotectin; ESR
Diabetes (Type 1 and 2)	HbA1c
Chronic liver disease, including Glycogen storage disorders and Transplant	Liver function tests and albumin level
Phenylketonuria	Serum Phe and tyrosine levels
Systemic lupus erythematosus	Antinuclear antibodies
Congenital heart disease and rhythm disorders	Exercise stress test; Echocardiography; ECG

Baseline readiness to transition will be assessed using the Trapeze Transition Readiness Checklist (which is designed by the Trapeze transition service (Available on Trapeze website at http://www.trapeze.org.au/content/checklist-young-people and attached in Additional file 2). The Trapeze Transition Readiness Checklist has not been validated, and to the the best of our knowledge, neither have any of the commonly used transition readiness questionnaires have been fully validated. A score of greater than 37 in the Trapeze Transition Readiness Checklist represents achievement of 70% task competency and would be defined as being “Transition Ready” by expert clinicians in practice.

Mental health risk factors will be assessed at baseline using the validated Kessler Psychological Distress scale (K-10) (Additional file 3). A K-10 score greater than 20 would be considered indicative of having mental health risk factors (possible mental disorder).

#### Engagement and retention with adult service

Successful engagement with the adult service at 6 months will be defined as confirmed attendance at least one appointment with the adult service by six months post discharge from paediatric care (i.e. after the last paediatric appointment). This will be considered as a *successful transfer* to adult services. This number is chosen because most chronic conditions have guidelines on the optimal number of specialist visits per year with a frequency varying from 3 to 6 months. Routine annual visits are less likely and will be recorded as such.

*Engagement* with the adult service will be assessed, with consent of the young person/parent, (provided at enrolment) via phone call to the young person and nominated adult service at 6 months post discharge from paediatric care. If the young person has not attended the service they were referred to, they will be asked if they are attending an alternate service and for consent to contact this service. Young people who cannot be contacted at 6 and 12 months post discharge from the paediatric service will be considered to have unsuccessful engagement and be a *failed transfer* to adult care.

*Retention* within the adult service will be defined as at least one confirmed attendance with the adult service in the preceding 6 months at 12 and 18 months follow up. These data will be collected, with consent, via phone call to the young person and the adult service to obtain information regarding attendance at the adult service at 12 and 18 months follow up. Young people who cannot be followed up at 12 and 18 months post discharge from the paediatric service will be considered to not be retained by the adult service (*failed retention*).

#### Measures of illness control

Deterioration of illness control will be assessed by looking at clinically utilised measures of illness control for chronic illnesses as specified in the Table 1 above. In the absence of empirical data, we have consulted with subspecialists within the paediatric services regarding commonly utilised measures of disease control and determination of clinical deterioration. Deterioration in illness control will be measured in a binary form: yes or no. Examples from commonly transitioning conditions are presented in Table 2. Measures of illness control will be obtained from the paediatric service at baseline, and the adult service and/or patient self-report during follow-up at 6, 12 and 18 months. Prevention of disease deterioration will be defined in a binary form: yes or no. Young people for whom information on markers of disease control from the adult service as defined above cannot be obtained, will be considered to have had a deterioration in their illness control.

**Table 2:** Example measures of disease control and definition of deterioration

Chronic illness	Measure of disease control	Definition of Deterioration
Cystic Fibrosis	FEV_1_	FEV1 decreased by ≥10%
Type 1 Diabetes Mellitus	HbA1c	Absolute decrease in HbA1C by ≥ 1% (e.g. 9% reducing to 8%)
Inflammatory Bowel Disease	CRP Hb	CRP ≥ 75% increase ≥ 10% fall from previous level
Cystic Fibrosis Inflammatory Bowel Disease	Weight Weight	Weight decreased by ≥5% Weight decreased by ≥5%
All Chronic illnesses	Hospitalisation	≥ 1 UNPLANNED hospitalisation related to chronic illness

The number of unplanned hospitalisations related to chronic illness will also be recorded.

#### Feedback about the TMApp

Participants will complete a semi-structured interview with a member of the research team (in person or via Skype or teleconference) at the end of the 12 month follow up period to provide feedback about ease of use, frequency and barriers in regard to the use of the TMApp (See Additional file 4).

Analytics (digital footprint) of participants’ interaction with the app will be also obtained. These analytics are de-identified, grouped data that will document how often the app is used, the frequency and duration of use of the app, time spent on the app and the most frequently used functions. There is a disclaimer for the TMApp on both the App store and Google play store to ensure only study participants download the application.

### Criteria for discontinuing or modifying allocated interventions

The intervention may be subject to modification in the following circumstances: to ensure optimal functioning of the TMApp (to address crashes; fix bugs; necessary updates as required by the application operating systems – Android and IOS) and if there are significant issues identified by the participant or the research team during the study.

### Strategies to improve adherence to interventions

A fortnightly reminder message (“Any events coming up?” “Time to check your TO DO list”) is incorporated into the TMApp to encourage engagement of the participants with the app. The messages are sent at 7 pm every other Sunday through the TMApp. This time was chosen as the day that the young people are least likely to have after-hours commitments.

### Relevant concomitant care permitted or prohibited during the trial

The Transition Mate application is in addition to and does not preclude the participants from any existing transition support and services.

### Outcomes

#### Primary outcome

- To measure engagement with adult services, we will review attendance of the participants at adult appointments at 6 months. Engagement is defined by at least one attendance to the adult service in the preceding 6 months at the 6 months follow-up. This is obtained from the adult service or patient self-report via study-specific questionnaires. (Questionnaires in Additional files) and will be supplemented by information from hospital databases. TMApp analytics will provide further information on the TMApp usage.

Time point: At 6 months after last clinic appointment at paediatric service.

#### Secondary outcomes

- To measure retention, we will review attendance of the participants at adult appointments at 12 and 18 months. Retention is defined by at least one further attendance to the adult service in the preceding 6 months at the 12 and 18 months follow-up. This is obtained from the adult service or patient self-report via study-specific questionnaires. (Questionnaires in Additional files 4, 5, 6) and will be supplemented by information from hospital databases.
- To understand change in illness control during the first 18 months post transition, we will review the changes in the markers of illness control from baseline. We will also assess the number of unplanned/ emergency hospital admissions of the participants after their last paediatric appointment. These are obtained from the adult service or patient self-report via study-specific questionnaires. (Questionnaires in Additional files 4, 5, 6) and will be supplemented by information from hospital databases.
- To understand if the use of the TMApp can assist with preventing deterioration of chronic illness control in young people in the 12 months post transition, we will analyse the changes in illness control with TMApp usage data. This will be obtained from TMApp analytics as well as adult service or patient self-report via study-specific questionnaires. (Questionnaires in Additional files 4, 5, 6) which will be supplemented by information from hospital databases.

Time point: At 6 months, 12 months (primary endpoint) and 18 months after last clinic appointment at the paediatric service.

### Participant timeline

**Table:** 

**Example procedures**	**Assessment/ Procedure**	Initial Assessment (*Penultimate or Final Paediatric appointment	**6 months**	**12 months**	**18 months**
	**Informed Consent** **(Additional file** **1****)**	**x**			
	**Demographic Information including standard transition care received** **Including information about Adult Service referred to** **(Additional file** **8****)**	**x**			
	**Measure of disease control**	**x**	**x**	**x**	**x**
	**Transition Readiness Assessment Questionnaire** **(Additional file** **2****)**	**x**			
	**Kessler Psychological Distress Scale (K-10)** **(Additional file** **3****)**	**x**			
	**Download and use TransitionMate app**	**x**	**x**	**x**	
	**Phone call or text message to young person re: attendance/engagement with adult service and measure of disease control** **(Additional files** **4****,** **5****,** **6****)**		**x**	**x**	**x**
	**Phone call or written request to adult service re: attendance/engagement with adult service and measure of disease control** **(Additional files** **4****,** **5****,** **6****)**		**x**	**x**	**x**
	**Semi-structured interview (face-face, Skype or Telephone) about effectiveness of TransitionMate app** **(Additional file** **4****)**			**x**	

### Sample size

#### Numbers required (power analysis)

There is limited empirical evidence on the effectiveness of current transition programs in regard to engagement with and retention in adult services or on rates of deterioration in illness control on which to base power calculations. In the diabetes literature, approximately 70% of adolescents will make some form of transition from paediatric to adult services [23, 24]. Using a predicted attrition rate of 30%, a sample size of 70 participants will be needed to obtain the minimum sample of 49 adolescents required for this study. A sample of 49 adolescents will have greater than or equal to 80% power at 5% one-sided alpha, to detect an increase in the rate of successful transition from paediatric to adult care from 70%.

### Recruitment

Participants will be recruited from the paediatric outpatient clinics within the Sydney Children’s Hospital Network (Sydney Children’s Hospital at Randwick and The Children’s Hospital at Westmead). Suitable patients aged 16 years and over with a chronic illness requiring transition to adult services in their final year of paediatric care/attending their penultimate paediatric appointment will be identified by the treating team or clinic staff and will be approached for recruitment to be part of the study by a member of the research team.

All clinical teams from the two tertiary children’s hospital across the network who may have potential study recruits will be contacted directly by the researchers. The researchers will present the TransitionMate study and application to the teams. Recruitment inclusion as well as exclusion criteria will be explained to the respective clinical teams. A contact person from each subspecialty will be identified to coordinate ongoing recruitment of patients who fit the recruitment inclusion criteria. The clinical team make the first approach and only when the patient has agreed to be approached by the researchers do the researchers contact them. The research team will discuss with the contact person from the respective clinical team as to the best avenue to approach the patients for recruitment. This may be in a different clinical setting including transition clinics, routine follow-up clinics and during planned admissions or clinical procedures. The research team will also coordinate with the hospital’s Trapeze transition services to identify potential recruits. The research team will provide ongoing updates and liaison with the contact person from each clinical team and transition services every 3 months. This will include prospective review of clinic lists to determine if there are patients who fulfil criteria for the study.

## Statistical methods

### Statistical methods for primary and secondary outcomes

The primary outcome as the percentage of adolescents who successfully engage and secondary outcomes of the percentage who are retained within the adult health care services will be described using a 90% exact binomial confidence interval. If the lower confidence limit is less than or equal to 70%, the TMApp would not be considered effective in terms of increasing rates of engagement and retention within the adult services.

Participant demographics, clinical characteristics and study outcomes will be described using standard methods, including frequencies and percentages for categorical variables, and mean, standard deviation, median, quartiles and range for continuous variables. Kaplan-Meier methods will be used for time-to-event variables. Study outcomes will be described in the total cohort as well as by chronic illness group, acknowledging that sub-group numbers will limit further analyses.

Linear, logistic or proportional hazards regression models will be used to examine predictors of study outcomes including TMApp use, age, gender, illness, transition readiness and mental health status in univariate models and models adjusted for potential confounders including other transition programs, use of other applications and deterioration in illness control. All effects will be presented with confidence intervals where possible.

### Interim analyses

Not planned as this is primarily an observational study of usage of an application, which does not have any direct adverse effects

### Methods for additional analyses (e.g. subgroup analyses)

Not applicable as we do not expect the sample size to be large enough for any subgroup analysis of specific chronic illnesses.

### Methods in analysis to handle protocol non-adherence and any statistical methods to handle missing data

Handling of non-adherence is not called for other than recording non-adherence data as an outcome.

Missing data will be managed as recording only. If data are missing and remain missing from any time point of collection the assumption will be that this represents non-engagement with the adult service.

## Discussion

This is likely to be the first trial of an application which has been developed as a generic tool to support transition from paediatric to adult health care. There is a paucity of literature considering the final active phase of transition. This is not surprising as this study is challenging given that the participants are moving to multiple care centres, young people change phones, the engagement in the study is likely to be a low priority and our functional application is competing with any number of attractive and engaging commercial applications which have the capacity to be rapidly modified by user feedback. The recruitment of adolescents to research studies has historically been challenging [27, 28]. This is particularly so with the transition phase as we are aiming to recruit these young people in their final year of paediatric care before they transition from paediatric to adult services. The diversity of the respective transition processes and challenges faced by the young people with chronic illnesses may also impede on our recruitment. Therefore, significant emphasis has been made by the researchers to inform, collaborate and maintain communication with the different clinical teams throughout the recruitment process. The identification of a contact person for each clinical team is an important step in this. Every effort will be made by the researchers to accommodate to the young people and clinical teams in order to minimise interference to their clinical care. No additional appointments are required for the recruitment process as the researchers will travel to meet the potential recruits at their planned clinics or admission as part of their routine medical care.

We have deliberately chosen a pragmatic approach for this trial in order to collect some very basic knowledge about usage and impact of applications in health care. This will be an informative study whatever the outcomes. Firstly, transition is a dynamic and multi-factorial process and our approach allows the best opportunity for a rapid recruitment during the final transition from paediatric to adult care period which in Australia occurs in the fourth quarter of the calendar year. Secondly, recruitment and retention is challenging for the adolescent age group, and it would be impossible to blind the recruitment which occurred in clinic spaces.]. Thirdly, mobile technology evolves rapidly and the TMApp may require regular updates and repair, or become outmoded. Fourthly, we do have some historical data and clinic data as a comparison group [20, 21]. The study is maximizing the opportunity to make some meaningful conclusions which will assist future studies and our own research in digital health care.

## Supplementary Information

**Additional file 1.** Participant Information Sheet/Consent Form.

**Additional file 2.** Transition_Readiness_Checklist

**Additional file 3.** Kessler Psychological Distress Scale (K-10)

**Additional file 4.** 12 month Follow Up

**Additional file 5.** 6 month Follow Up

**Additional file 6.** 18 month Follow up

**Additional file 7.** Letter to Adult Service

**Additional file 8.** Study Patient Demographics questionnaire

## Data Availability

The datasets used and/or analysed during the current study are available from the corresponding author on reasonable request. ANCILLARY DATA. There will be no ancillary data collected by the current project. USE OF DATA AND PUBLICATIONS POLICY/ AVAILABILITY OF DATA AND MATERIALS. The information will be owned by the principal investigators of the project. Results will be submitted for publication in appropriate journals. The datasets used and/or analysed during the current study are available from the corresponding author on reasonable request. Participants have a right to receive feedback about the overall summary results of the study. Their choice to receive feedback will be ascertained by ticking the feedback box on the consent form. This feedback will be in the form of a summary that will be posted or emailed to the nominated postal or email address. The feedback will be received after the study is finished, and all results will be de-identified.

## Appendices

Table 3.

**Table 3:** List of Attachments included

Appendix number	Document Name	Developed for this study	Version Number	Date of version approved by ethics
1	Participant information and consent form (PICF)	Yes	2.2	18/12/2017
2	Transition Readiness Checklist (TRC)	No	1.0	27/09/2017
3	Kessler Psychological Distress Scale (K-10)	No	1.0	27/09/2017
4	TransitionMate 12 structure questionnaire	Yes	3.0	01/07/2019
5	TransitionMate 6 months structure questionnaire	Yes	3.0	01/07/2019
6	TransitionMate 18 month structure questionnaire	Yes	2.1	30/10/2017
7	Letter to adult service requesting information	Yes	1.0	27/09/2017
8	TransitionMate Study Patient Demographics Questionnaire	Yes	1.0	27/09/2017

## Abbreviations

TMApp: TransitionMate application
FEV_1_: Forced expiratory volume in one second
HbA1c: Glycated haemoglobin
SMS: Short message service
ACI: Agency for Clinical Innovation
TSH: Thyroid stimulating hormone
MRI: Magnetic resonance imaging
GMFCS: Gross Motor Function Classification System
CRP: C-reactive protein
ESR: Erythrocyte sedimentation rate
LFTS: Liver function tests
BPM: Beats per minute
ECG: Electrocardiogram
SCHN: Sydney Children’s Hospital Network
Hb: Haemoglobin
T1DM: Type 1 diabetes mellitus
CF: Cystic fibrosis
