# Supplementary material for: Study protocol: a pragmatic trial reviewing the effectiveness of the TransitionMate mobile application in supporting self-management and transition to adult healthcare services for young people with chronic illnesses
Source: BMC Health Serv Res. 2022 Nov 29;22:1443. doi: 10.1186/s12913-022-08536-8 (PMC9706969; doi:10.1186/s12913-022-08536-8)
Supplement: Supplementary file 1 — Additional file 1. Participant Information Sheet/Consent Form. [file 12913_2022_8536_MOESM1_ESM.doc]

**Participant Information Sheet/Consent Form**

| **Title** | TransitionMate: A mobile app to support self-management and transition in young people with chronic illness |
| --- | --- |
| **Short Title** | TransitionMate: A mobile app to support transition in young people with chronic illness |
| **Coordinating Principal Investigator/ Principal Investigator** | Professor Katharine Steinbeck |
| **Associate Investigator(s)** | A/Prof Susan Towns, Dr Jane Ho, Dr Vathsala Agarwalla, Dr Yiselle Ilene Virella-Perez, *Dr Vhari Forsyth (to be added in 2018)* |
| **Location** | The Children’s Hospital at Westmead  Sydney Children’s Hospital |

This Participant Information Statement tells you about the research study. Knowing what is involved will help you decide if you would like to take part in this study. Please read this sheet carefully and ask questions about anything that you don’t understand or want to know more about.

Participation in this research study is voluntary. It is up to you whether you wish to take part or not.

1. **Introduction**

Thank you for considering taking part in the “**TransitionMate: A mobile app to support transition in young people with chronic illness**” research project that is running through the Department of Adolescent Medicine at The Children’s Hospital at Westmead. The research project aims to find out if the use of the TransitionMate app during transition from the Children’s Hospital to adult services can help you link up with adult services and prevent any loss in control of you medical condition.

If you decide to take part in the research project, you will be asked to sign the consent section. By signing it you are telling us that you:

• Understand what you have read

• Consent to taking part in the research project

• Consent to the researchers contacting you and the adult service you have been referred to at 6,12,and 18 months after the study starts

• Consent to the use of your health and demographic information as specified in this Participant Information Sheet and Consent form.

You will be given a copy of this Participant Information and Consent Form to keep.

**2 What is the purpose of this research?**

TransitionMate is a mobile phone application (app) that we have developed to support self-management and transition from paediatric to adult care in young people with chronic illness.

The TransitionMate app has a number of important functions to help you with managing your health condition, such as to-do lists and reminders (for medication, treatment or appointments), calendar, recording and graphing measures of illness control ( tracking health measures), images (to store clinic letters, prescriptions or results), contact details (for doctors or clinics) and tracking your mood and energy levels.

The app requires that you input your own health related data, and we anticipate that initial set up of the app and input of health related data which we will help you with should take no more than 45 minutes. All health related data is stored locally on your mobile device and does not get uploaded onto a server or communicated with the app developers or any health care providers. None of the information that you input into the app is relayed back to the app developer/health professionals/research team or anybody else. You are free to share your information with relevant health professionals if you choose.

The aim of this study is to find out if the use of the TransitionMate app during transition to adult care will help you link up with adult services, and prevent deterioration in measures of illness control relevant to you condition.

1. **Who can participate in this study?**

Any young person age 16 years or over who has a chronic illness and is a current patient within the Sydney Children’s Hospital Network (Sydney Children’s Hospital Randwick and The Children’s Hospital Westmead) can participate. Young people aged 16 years and over with chronic illness in their final year of paediatric care and who require transition to adult services will be identified by their treating team or clinic staff and will be invited to participate in the study by a member of the research team.

**4 What does the participant have to do?**

You will be approached in your final year prior to transition to adult services at The Sydney Children’s Hospital Network, preferably at your second last paediatric appointment if possible. If you consent to the study, we will request you complete a questionnaire with some information about yourself (including DOB, age, sex, ethnicity, diagnosis, contact details). We will also collect relevant measures of illness control from your medical record. Your treating paediatric specialist will decide with you which adult specialist or service you will see, and we will ask for consent to contact both you and your adult specialist in the future (at 6, 12 and 18 months).

We will give you information on how to access and use the “TransitionMate” app from either a weblink that will be sent to you, or by downloading if (for free) from the App store and show you how to use it. We will ask you to use the app during the 12 month period after you are discharged from the Children’s hospital.

We will then ask you to complete two questionnaires at the clinic – The Transition Readiness Checklist and the Kessler-10 (K-10) checklist, that help us understand your chronic illness knowledge and care, readiness for transition and any mental health issues that can affect successful transition. This will all take approximately 35 minutes.

The trial co-ordinators will contact you at 6, 12, 18 months to see if you have seen your team in the adult service, how your illness control is going and if you have been in admitted to hospital because of your illness. This contact would be via your preferred contact email/phone/SMS. At the 12 month follow-up we will also ask you about the usability and effectiveness of the TransitionMate app. At 12 months, the research team will also obtain analytics (a digital footprint) of how you and other participants interact with the app. This will help the research team understand if you used the app, how often you used the app, how long you used the app for, how much time you spent on the app and what were your most used functions. **All of this information will be de-identified, which means we cannot identify who you are from the data analytics.** As mentioned previously, none of the information that you input into the app is relayed back to the app developers, health professionals or research team.

This is what you consent to if sign Part A of the consent form

The investigators would also like your permission to contact the adult specialist to whom you have been referred to after leaving the paediatric clinic to find out how many times you have visited them and how your illness control is going.

This is what you consent to if you sign Part B of the consent form

You can sign Part A and Part B, or Part A only.

**5 Do I have to take part in this research project?**

Your participation in any research project is voluntary. If you do not wish to take part, you do not have to. If you decide that you want to take part and later change your mind, you are free to withdraw from the project at any stage. If you do decide that you can take part, you will be given this Participant Information and Consent Form to sign.

Your decision whether you can or cannot take part, or take part and then withdraw, will not affect your routine treatment, relationship with those treating you or your relationship with the clinic/hospital.

**6 What are the possible benefits of taking part?**

Whilst we intend that this research study will improve our knowledge of transition from paediatric to adult care, of the usefulness of a mobile phone app to support transition and may also help us improve the transition process in the future, we cannot guarantee or promise that you will receive any direct benefits from being in the study.

**7 What are the possible risks and disadvantages of taking part?**

The risks of this research are very low as it does not involve any treatments or medications. There are questionnaires that you will be asked to complete and some questions asked by the doctor and if you agree, we will also contact the adult service. The risk of psychological and physical distress is considered to be low. If you were to become distressed, we could provide assistance and referral as required. **The TransitionMate App is not passcode protected. If your phone is accessed without your permission anything contained in the App may also be accessed and viewed. We recommend you passcode protect your phone and that all identifiable records be de-identified prior to uploading.** At the 12 month follow-up we may identify young people who have not engaged with adult services as recommended by their paediatric team. If this happens for you, we would ask your permission to contact Transition services to help you link in with an adult service.

**8 What if the participant withdraws from this research project?**

If you decide to withdraw from this research project, please notify a member of the research team before you withdraw. If you do withdraw your consent during the research project, the study doctor and relevant study staff will not collect additional personal information from you, although personal information already collected will be kept to make sure that the results of the research project can be measured properly and to comply with law. You should be aware that data collected up to the time you withdraw may form part of the research project results in de-identified form. If you do not want us to do this, you must let us know before you join the research project.

**9 Could this research project be stopped unexpectedly?**

This research project may be stopped unexpectedly for a variety of reasons. These may include funding issues or any other unforeseen reasons*.*

**10 What will happen to information about the participants?**

Your information will only be used for the purpose of this research project and it will be treated as confidential and securely stored. Information will only be disclosed with your permission, except as required by law. Information may be obtained from your health records held at this and other health services for the purpose of this research. By signing the consent form you agree to the research team accessing health records if these are relevant to your participation in this research project.

All data will have all personal details removed before being stored. Documents will be kept highly confidential and will remain in a locked filing cabinet within the Academic Department of Adolescent Medicine (The Children’s Hospital at Westmead), Westmead Hospital. Digital data will be on a password-protected database on a restricted access network drive. All data will be stored for 7 years after completion of the study, (or until you reach 25 years). After this time, hard copy data will be destroyed and computer data will be erased.

**11 How do you intend to publish or disseminate the results?**

It is anticipated that the results of this research project will be published and/or presented in a variety of forums including lectures, conferences and scientific journals. In order for the wider community to benefit from the project, we plan to produce reports and/or articles that are publicly available. We will ensure that in any publication or presentation of these reports, information is presented in a non-identified and summary form, so that individual participants cannot be identified. Your privacy will be protected at all times.

1. **Will we be told the results of the study?**

You have a right to receive feedback about the overall summary results of this study. You can tell us that you wish to receive feedback by ticking the feedback box on the consent form. This feedback will be in the form of a summary that will be posted or emailed to your nominated contact address. You will receive this feedback after the study is finished.

**13 What if we have a complaint or any concerns about the study?**

All research in Australia involving humans is reviewed by an independent group of people called a Human Research Ethics Committee (HREC). The ethical aspects of this research project have been approved by the HREC of Sydney Children’s Hospital Network. This project will be carried out according to the *National Statement on Ethical Conduct in Human Research (2007)*. This statement has been developed to protect the interests of people who agree to participate in human research studies.

If you have concerns or complaints about the project or the use of your personal and health information you should contact the Executive Officer from the Human Research Ethics Committee that has also approved this project.

**14 Further information and who to contact**

The person you may need to contact will depend on the nature of your query. If you want any further information concerning this project, you can contact the principal study doctor (Prof Kate Steinbeck) on 02 9845 2507 or any of the following people:

**Clinical contact person**

| Name | Dr Vathsala Agarwalla |
| --- | --- |
| Position | Marie Bashir Clinical Research Fellow in Adolescent Health |
| Telephone | 02 9845 2290 |
| Email | Vathsala.Agarwalla@health.nsw.gov.au |

If you have any complaints about any aspect of the project, the way it is being conducted or any questions about being a research participant in general, then you may contact:

**Reviewing HREC approving this research** **and HREC Executive Officer details**

| Reviewing HREC name | SCHN Human Research Ethics Committee |
| --- | --- |
| HREC Executive Officer | Ms Asra Gholami |
| Telephone | 98453066 |
| Email | SCHN-Ethics@health.nsw.gov.au |

**Consent Form – PARTICIPANT CONSENT**

| **Title** | TransitionMate: A mobile app to support self-management and transition in young people with chronic illness |
| --- | --- |
| **Coordinating Principal Investigator/ Principal Investigator** | Professor Katharine Steinbeck |
| **Associate Investigator(s)** | A/Prof Susan Towns, Dr Jane Ho, Dr Vathsala Agarwalla, Dr Yiselle Ilene Virella-Perez |
| **Location** | The Children’s Hospital at Westmead  Sydney Children’s Hospital |

**Declaration by Participant**

I have read the Participant Information Sheet or someone has read it to me in a language that I understand. I understand the purposes, procedures and risks of the research described in the project. I have had an opportunity to ask questions and I am satisfied with the answers I have received. I freely agree to participating in this research project as described and understand that I am free to withdraw at any time during the project without this affecting my future health care. I understand that all data will be in de-identified and confidential form.

If you are over18 years old, you can sign the consent form yourself. If you are younger than18 years old, we will require your parent/guardian’s consent for you to participate

I understand that I will be given a signed copy of this document to keep.

I CONSENT TO:

PART A Please Tick if you consent

- Participation in the research study as specified above including providing demographic information, downloading and using the TransitionMate app, and answering questionnaires
- The researchers contacting me at 6, 12 and 18 months for information about engagement with the adult service, markers of illness control and utility of the “TransitionMate” app

PART B Please Tick if you consent

- The researchers contacting the adult service that I have been referred to (name) ______________________________ at 6, 12 and 18 months and for the adult service (name) _______________________ to provide information to the researchers about:
  - My attendance at the adult service
  - Standard measures of chronic illness control __________ (specify)

Receiving feedback about the outcome of this project

|  | | | | | | | |
| --- | --- | --- | --- | --- | --- | --- | --- |
| Name |  | |  | |  |  |  |
|  | | | | | | | |
| Signature |  |  | | Date | |  |  |
|  | | | | | | | |

Parental Consent if <18 years old

|  | | | | | | | |
| --- | --- | --- | --- | --- | --- | --- | --- |
| Name of Parent/Guardian  (  Signature Date |  | |  | |  |  |  |
|  | | | | | | | |
| Signature |  | Date | |  | |  |  |

|  | | | | | | |
| --- | --- | --- | --- | --- | --- | --- |
|  | Name of Witness* to Participant signature (please print) | |  | | |  |
|  |  |  |  | | |  |
|  | Signature |  | | Date |  |  |
|  | | | | | | |

* Witness is not to be the investigator, a member of the study team or their delegate. In the event that an interpreter is used, the interpreter may not act as a witness to the consent process. Witness must be 18 years or older.

**Declaration by Study Doctor/Senior Researcher^†^**

I have given a verbal explanation of the research project, its procedures and risks and I believe that the participant /parent/guardian of the participant has understood that explanation.

|  | | | | | | |
| --- | --- | --- | --- | --- | --- | --- |
|  | Name of Study Doctor/  Senior Researcher^†^ (please print) | |  | | |  |
|  | | | | | |  |
|  | Signature |  | | Date |  |  |
|  | | | | | | |

^†^ A senior member of the research team must provide the explanation of, and information concerning, the research project.

Note: All parties signing the consent section must date their own signature.

**Form for Withdrawal of Participation – PARTICIPANT**

| **Title** | TransitionMate: A mobile app to support self-management and transition in young people with chronic illness |
| --- | --- |
| **Coordinating Principal Investigator/ Principal Investigator** | Professor Katharine Steinbeck |
| **Associate Investigator(s)** | A/Prof Susan Towns, Dr Jane Ho, Dr Vathsala Agarwalla, Dr Yiselle Ilene Virella-Perez |
| **Location** | The Children’s Hospital at Westmead  Sydney Children’s Hospital |

**Declaration by Participant**

I wish to withdraw myself from participation in the above research project and understand that such withdrawal will not affect my routine treatment, relationship with those treating them or relationship with the hospital.

|  |  | |  | | | |  |
| --- | --- | --- | --- | --- | --- | --- | --- |
|  | Name |  | | | | |  |
|  |  |  | | | | |  |
|  | Signature | |  | | Date |  |  |
|  | If  If | |  | |  |  |  |
|  | Name of Parent/Guardian (under 16 only | | |  | | |  |
|  |  | | |  | | |  |
|  | Signature of Parent/Guardian | |  | | Date |  |  |
|  | | | | | | | |

In the event that the participant’s decision to withdraw is communicated verbally, the Study Doctor/Senior Researcher will need to provide a description of the circumstances below.

|  |
| --- |

**Declaration by Study Doctor/Senior Researcher^†^**

I have given a verbal explanation of the implications of withdrawal from the research project and I believe that the parent/guardian of the participant has understood that explanation.

|  | | | | | | |
| --- | --- | --- | --- | --- | --- | --- |
|  | Name of Study Doctor/  Senior Researcher^†^ (please print) | |  | | |  |
|  | | | | | |  |
|  | Signature |  | | Date |  |  |
|  | | | | | | |

^†^ A senior member of the research team must provide the explanation of, and information concerning, withdrawal from the research project.

Note: All parties signing the consent section must date their own signature.
