# Supplementary material for: Study protocol: a pragmatic trial reviewing the effectiveness of the TransitionMate mobile application in supporting self-management and transition to adult healthcare services for young people with chronic illnesses
Source: BMC Health Serv Res. 2022 Nov 29;22:1443. doi: 10.1186/s12913-022-08536-8 (PMC9706969; doi:10.1186/s12913-022-08536-8)
Supplement: Supplementary file 2 — Additional file 2. Transition_Readiness_Checklist [file 12913_2022_8536_MOESM2_ESM.pdf]

TRANSITION READINESS CHECKLIST

Use this checklist to identify the skills you already have, and the areas where you may need to increase your knowledge to help you prepare for transition.

Date: ____ **MRN:** ____ **Name:** ____ **Sex:** ____ **DOB:** ____

Home Phone: ____ **Mobile:** ____ **Email:** ____

Interpreter required: ____ **Preferred language:** ____ **Mailing address:** ____

	I'm on top of this	I need to work on this	I have no idea	N/A
1. I know the names of my medications and what they are for.				
2. I have allergies and I know how to manage them.				
3. I can confidently name and explain my medical condition and treatment plan.				
4. I am responsible for remembering and administering my medications.				
5. I am aware of any side effects of the medications I take.				
6. I am responsible for getting my prescriptions.				
7. I know the equipment I need for treatment and what it is used for.				
8. I am familiar with the tests that I have regularly and why I need to have them.				
9. I can make or reschedule my own appointments.				
10. I know who I can direct health questions to and I feel comfortable asking.				
11. I can attend appointments without my parent/ guardian present.				
12. I have a GP and feel comfortable with.				

TICK THE BOXES

trapeze
a supported leap into adult health

The Sydney children's Hospitals Network
care, advocacy, research, education

TRANSITION READINESS CHECKLIST cont.

	I'm on top of this	I need to work on this	I have no idea	N/A
13. I know what to do when I become unwell.				
14. I know who to contact if I need help.				
15. I know where/how to get information about peer support programs.				
16. I know about resources that offer support for young people like me.				
17. I understand my rights to privacy and my role in decision making.				
18. I know where to get information about sexual health, drugs, alcohol and stress.				
19. I understand what transition means.				
20. I have been given information about the adult service and I feel comfortable about the choice.				
21. I am actively involved in my transition.				
22. I have my own Medicare card.				
23. I have my own Health care card.				
24. I know my private health insurance details.				
25. I know the names and contact information of the people I'm seeing in the adult service.				
26. I have visited the adult service I am transitioning to.				
27. I have attended my first appointment for my new health service.				

Comments:

____

____

____

TICK THE BOXES
