# Supplementary material for: Study protocol: a pragmatic trial reviewing the effectiveness of the TransitionMate mobile application in supporting self-management and transition to adult healthcare services for young people with chronic illnesses
Source: BMC Health Serv Res. 2022 Nov 29;22:1443. doi: 10.1186/s12913-022-08536-8 (PMC9706969; doi:10.1186/s12913-022-08536-8)
Supplement: Supplementary file 3 — Additional file 3. Kessler Psychological Distress Scale (K-10) [file 12913_2022_8536_MOESM3_ESM.pdf]

Kessler Psychological Distress Scale (K10)
Source: Kessler R. Professor of Health Care Policy, Harvard Medical School, Boston, USA.

This is a 10-item questionnaire intended to yield a global measure of distress based on questions about anxiety and depressive symptoms that a person has experienced in the most recent 4 week period.

<u>Why use the K10</u>
The use of a consumer self-report measure is a desirable method of assessment because it is a genuine attempt on the part of the clinician to collect information on the patient's current condition and to establish a productive dialogue. When completing the K10 the consumer should be provided with privacy.
(Information sourced from the NSW Mental health Outcomes and Assessment Training (MH-OAT) facilitator's Manual, NSW Health Department 2001)

<u>How to administer the questionnaire</u>
As a general rule, patients who rate most commonly "Some of the time" or "All of the time" categories are in need of a more detailed assessment. Referral information should be provided to these individuals. Patients who rate most commonly "A little of the time" or "None of the time" may also benefit from early intervention and promotional information to assist raising awareness of the conditions of depression and anxiety as well as strategies to prevent future mental health issues.

(Information sourced from the NSW Mental health Outcomes and Assessment Training (MH-OAT) facilitator's Manual, NSW Health Department 2001)

K10 Test

These questions concern how you have been feeling over the past 30 days. Tick a box below each question that best represents how you have been .

**1. During the last 30 days, about how often did you feel tired out for no good reason?**				
1. None of the time	2. A little of the time	3. Some of the time	4. Most of the time	5. All of the time

**2. During the last 30 days, about how often did you feel nervous?**				
1. None of the time	2. A little of the time	3. Some of the time	4. Most of the time	5. All of the time

**3. During the last 30 days, about how often did you feel so nervous that nothing could calm you down?**				
1. None of the time	2. A little of the time	3. Some of the time	4. Most of the time	5. All of the time

**4. During the last 30 days, about how often did you feel hopeless?**				
1. None of the time	2. A little of the time	3. Some of the time	4. Most of the time	5. All of the time

**5. During the last 30 days, about how often did you feel restless or fidgety?**				
1. None of the time	2. A little of the time	3. Some of the time	4. Most of the time	5. All of the time

**6. During the last 30 days, about how often did you feel so restless you could not sit still?**				
1. None of the time	2. A little of the time	3. Some of the time	4. Most of the time	5. All of the time

**7. During the last 30 days, about how often did you feel depressed?**				
1. None of the time	2. A little of the time	3. Some of the time	4. Most of the time	5. All of the time

**8. During the last 30 days, about how often did you feel that everything was an effort?**				
1. None of the time	2. A little of the time	3. Some of the time	4. Most of the time	5. All of the time

**9. During the last 30 days, about how often did you feel so sad that nothing could cheer you up?**				
1. None of the time	2. A little of the time	3. Some of the time	4. Most of the time	5. All of the time

**10. During the last 30 days, about how often did you feel worthless?**				
1. None of the time	2. A little of the time	3. Some of the time	4. Most of the time	5. All of the time

Scoring

FOR DOCTOR'S EYES ONLY

This is a questionnaire for patients to complete. It is a measure of psychological distress. The numbers attached to the patients 10 responses are added up and the total score is the score on the Kessler Psychological Distress Scale (K10). Scores will range from 10 to 50. People seen in primary care who

* score under 20 are likely to be well
* score 20-24 are likely to have a mild mental disorder
* score 25-29 are likely to have moderate mental disorder
* score 30 and over are likely to have a severe mental disorder

13% of the adult population will score 20 and over and about 1 in 4 patients seen in primary care will score 20 and over. This is a screening instrument and practitioners should make a clinical judgement as to whether a person needs treatment.
Scores usually decline with effective treatment. Patients whose scores remain above 24 after treatment should be reviewed and specialist referral considered.

References:
Kessler, R.C., Andrews, G., Colpe, .et al (2002) Short screening scales to monitor population prevalences and trends in non-specific psychological distress. **Psychological Medicine, 32**, 959-956.

Andrews, G., Slade, T (2001). Interpreting scores on the Kessler Psychological Distress Scale (k10). **Australian and New Zealand Journal of Public Health, 25,** 494-497.
