# Supplementary material for: Study protocol: a pragmatic trial reviewing the effectiveness of the TransitionMate mobile application in supporting self-management and transition to adult healthcare services for young people with chronic illnesses
Source: BMC Health Serv Res. 2022 Nov 29;22:1443. doi: 10.1186/s12913-022-08536-8 (PMC9706969; doi:10.1186/s12913-022-08536-8)
Supplement: Supplementary file 4 — Additional file 4. 12 month Follow Up [file 12913_2022_8536_MOESM4_ESM.docx]

**TransitionMate 12 month Follow-up Structured Interview**

**Participant Number:**

**Date:**

Hello, my name is _________ a researcher from the Academic Department of Adolescent Medicine. As you may remember, you kindly agreed to help us out with the “TransitionMate: A mobile phone app to support self-management and transition of young people with chronic illness” study. When you consented to the study, you agreed to allow us to contact you now to see how your transition to adult services is going.

1. Have you seen your adult specialist / adult service __________ (insert name) in the last 6 months: YES/NO
   1. If yes, when did you see them?
   2. If no, do you have an appointment scheduled with them? YES/NO
      - - 1. If yes, when is it?
          2. If no, why not?

Have you decided to see another service? If so, where/who/why?

1. What was your most recent measure of chronic illness control? *(if indicated for condition, interviewer to specify the appropriate measure of disease control)*
   1. When was it done?
   2. Do you know the result? If so, can you please tell me?
2. Have you needed to go to hospital (i.e. for an unplanned hospital admission) because of your chronic illness in the last 6 months? YES/NO
   1. If yes, how many times?

Thank you for giving us the opportunity to find out how you are going. We will be in contact again in another 6 months’ time for an update.

Other:

- Not contactable
- Declined interview
- Requested to withdraw

**TransitionMate Usability Questionnaire Interview (12 Months)**

Over the last 12 months, have you used the TransitionMate app? YES/NO

- If yes, how frequently have you been using Transition Mate in the last month?
- Once or more per day
- Once or more per week
- Once or more per month
- Never

Which of the following features have you used frequently?

(You may choose more than one)

1. To do list Y/N
2. Measures Y/N
3. Contact Y/N
4. Medication Y/N
5. Images Y/N
6. Mood Y/N

- If no - why not? (*No further questions)*

Functionality:

- - 1. What was your favourite function(s) and why?
    2. Are there any functions that are missing or would be useful to include? YES/NO If yes, what?

Usability:

1. Was TransitionMate easy to use? YES/NO -> Why?
2. Would you recommend TransitionMate to other young people with chronic conditions? YES/NO -> Why?
3. On a scale of 1-10 (1 = not useful, 10 = most useful) how would you rate the usefulness of the TransitionMate app in helping your transition from paediatric to adult care and self-management skills?
