# Supplementary material for: Study protocol: a pragmatic trial reviewing the effectiveness of the TransitionMate mobile application in supporting self-management and transition to adult healthcare services for young people with chronic illnesses
Source: BMC Health Serv Res. 2022 Nov 29;22:1443. doi: 10.1186/s12913-022-08536-8 (PMC9706969; doi:10.1186/s12913-022-08536-8)
Supplement: Supplementary file 7 — Additional file 7. Letter to Adult Service [file 12913_2022_8536_MOESM7_ESM.docx]

Date:

Dear _______________________

Re: Name:

Date of Birth:

___________________________________ has agreed to participate in our “TransitionMate: A mobile app to support transition in young people with chronic illness” research project that is running through Department of Adolescent Medicine at The Children’s Hospital at Westmead within the Sydney Children’s Hospital Network

As part of this project _________________________has consented to the research team contacting you / your service at 6, 12 and 18 months post transition from paediatric to adult care for further information around:

1. Number of visits to your service in the last six months
2. Most recent measures of illness control

Please see the attached consent form signed by _____________________ allowing us to contact your service and obtain the above information. We will make contact with your service via telephone to ascertain the above information.

This research project has been reviewed by the SCHN Human Research Ethics Committee (HREC).

Our HREC Approval number is ________________

Should you have any questions or concerns, please feel free to contact the Marie Bashir Research Fellow in Adolescent Health at The Children’s Hospital Westmead on 98452290.

Thank you for your support with our research, to improve the health of young people.

Kind regards,

Professor Kate Steinbeck

Principal Investigator

Foundation Chair in Adolescent Medicine

Adolescent Physician and Endocrinologist, The Children’s Hospital at Westmead
