# Supplementary material for: Study protocol: a pragmatic trial reviewing the effectiveness of the TransitionMate mobile application in supporting self-management and transition to adult healthcare services for young people with chronic illnesses
Source: BMC Health Serv Res. 2022 Nov 29;22:1443. doi: 10.1186/s12913-022-08536-8 (PMC9706969; doi:10.1186/s12913-022-08536-8)
Supplement: Supplementary file 8 — Additional file 8. Study Patient Demographics questionnaire [file 12913_2022_8536_MOESM8_ESM.docx]

**TransitionMate Study Patient Demographics**

| **CHECKLIST**  **Consent**  **Demographics form with contact details**  **Unique Phone Identifier obtained**  **Download TransitionMate App / Instructions Given**  **Kessler K-10**  **Transition Readiness Checklist** | **□**  **□**  **□**  **□**  **□**  **□** |
| --- | --- |

**Participant Number:**

**Date:**

AFFIX STICKER

**Name:**

**DOB:**

**MRN:**

**Age:**

**Gender:**

**Diagnosis:**

**Ethnicity:**

**SCHN Treating Team and Primary Doctor:**

**Baseline Illness control measure and Date Done:**

**Adult Doctor / Adult Service Referred to:**

**Unique Phone Identifier:**

**Contact Details (Young Person)**

**Email:**

**Telephone:**

**Unique Phone Identifier:**

**Contact Details (Parents):**

**Email:**

**Telephone:**

**Input from:**

**Trapeze Y □ N □**

**ACI Y □ N □**

**Joint Adult/Paediatric Transition Clinic: Y □ N □**
